# Development and Differentiation of Midbrain Dopaminergic Neuron: From Bench to Bedside

**DOI:** 10.3390/cells9061489

**Published:** 2020-06-18

**Authors:** Mengmeng Wang, King-Hwa Ling, Jun Jie Tan, Cheng-Biao Lu

**Affiliations:** 1 Department of Neurobiology and Physiology, Xinxiang Medical University, Xinxiang 453003, Henan, China; wangmeng3589@student.usm.my; 2 The International-Joint Lab for Non-invasive Neural Modulation/Key Laboratory for the Brain Research of Henan Province, Xinxiang Medical University, Xinxiang 453003, Henan, China; 3 Advanced Medical and Dental Institute, University Sains Malaysia, Bertam 13200, Kepala Batas, Penang, Malaysia; 4 Department of Biomedical Sciences, Faculty of Medicine Universiti Putra Malaysia, Seri Kembangan 43400 Selangor, Malaysia; lkh@upm.edu.my; 5 Department of Genetics, Harvard Medical School, Boston, MA 02115, USA

**Keywords:** Parkinson’s disease, midbrain dopaminergic neuron, pluripotent stem cells, differentiation, neurodevelopment, small molecules

## Abstract

Parkinson’s Disease (PD) is a neurodegenerative disorder affecting the motor system. It is primarily due to substantial loss of midbrain dopamine (mDA) neurons in the substantia nigra pars compacta and to decreased innervation to the striatum. Although existing drug therapy available can relieve the symptoms in early-stage PD patients, it cannot reverse the pathogenic progression of PD. Thus, regenerating functional mDA neurons in PD patients may be a cure to the disease. The proof-of-principle clinical trials showed that human fetal graft-derived mDA neurons could restore the release of dopamine neurotransmitters, could reinnervate the striatum, and could alleviate clinical symptoms in PD patients. The invention of human-induced pluripotent stem cells (hiPSCs), autologous source of neural progenitors with less ethical consideration, and risk of graft rejection can now be generated in vitro. This advancement also prompts extensive research to decipher important developmental signaling in differentiation, which is key to successful in vitro production of functional mDA neurons and the enabler of mass manufacturing of the cells required for clinical applications. In this review, we summarize the biology and signaling involved in the development of mDA neurons and the current progress and methodology in driving efficient mDA neuron differentiation from pluripotent stem cells.

## 1. Introduction

**Figure 1:**
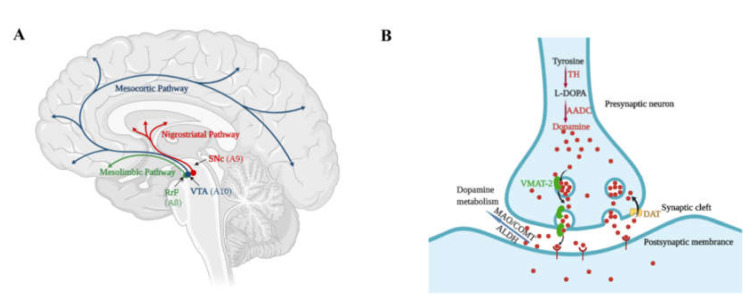
Dopamine pathway and dopamine neurotransmitters in the brain: (**A**) Dopaminergic pathways in the brain. mDA neurons are located in three distinct nuclei, the retrorubral field (RrF or A8 area), the substantia nigra pars compacta (SNc or A9 area), and the ventral tegmental area (VTA or A10 area). SNc mDA neurons project to the dorsal striatum via the nigrostriatal pathway. The VTA and RrF mDA neurons project to ventral striatum and prefrontal cortex forming the mesocortical and mesolimbic dopaminergic system. (**B**) The biosynthesis and metabolism of dopamine neurotransmitters. (Figures were created using BioRender.com).

Parkinson’s Disease (PD) is characterized by decreased nigrostriatal dopaminergic innervation due to the degeneration of dopaminergic neurons in substantia nigra pars compacta (SNc) A9 area (Figure 1A); the primary neurons constitute the nigrostriatal pathway [1,2]. This creates an imbalance in striatal-pallidal and pallido-thalamic output pathways and thus contributes to major motor dysfunctions. It is the second most common progressive neurodegenerative disorder after Alzheimer’s Disease and accounted for 6.1 million patients worldwide in 2016 [3]. The disease is known to strongly associate with aging as a result of increasing life expectancy. PD is rather uncommon in patients below 50 years, and most of the diagnosed PD patients have an age between 85 to 89 years [3]. Although the majority of PD cases are sporadic, about 15% of PD patients globally have a family history, some of which are related to mutations in the *LRRK2, PARK7, PINK1, PRKN*, or *SNCA* and *PRKN* genes [4]. 

As mentioned earlier, PD is caused by the degeneration of a specific mDA neuronal subtype in the SNc A9 area (Figure 1A). This is unlike the dopaminergic neuronal subtype that presents in the retrorubral field (RrF) A8 area and ventral tegmental (VTA) A10 area that constitutes the mesolimbic and mesocortical dopaminergic pathway. The loss of dopaminergic neurons in the SNc of nearly 30% with a 50–60% decrease of dopamine secretion in the corpus striatum is common in the majority of PD patients with the onset of motor dysfunctions [5,6], suggesting high severity of disease progression even at the first initial diagnosis. One of the pathological hallmarks of PD is the presence of Lewy bodies, a dense, spherical inclusion made of α-synuclein aggregates that present in the soma of neuronal cells, and the Lewy neurites, which are the abnormal α-synuclein clustering deposited in the axons. 

PD patients are diagnosed mainly based on clinical symptoms, including motor symptoms and non-motor symptoms. The cardinal motor symptoms of PD include bradykinesia, tremor, and rigidity, whereas non-motor characteristics include cognitive deterioration and other psychological problems such as sleep behavior disorder, depression, or anxiety [7]. Non-motor symptoms and complications, such as neuropsychiatric or neurobehavioral problems, autonomic dysfunction, and sensory problems, result from multiple neurotransmitter deficiencies in the central and peripheral nervous systems [8]. Non-motor symptoms may eventually become chief complaints and therapeutic challenges in advanced stages of PD. Nonetheless, studies have shown that some motor symptoms observed in PD, like postural instability and walking/gait problems, are mostly secondary to degeneration of non-dopaminergic pathways and significantly contribute to impairment and disability in advanced PD patients [8,9,10].

## 2. Current Treatment

The pharmacological approach is still the main primary treatment strategy for PD patients to alleviate or control motor symptoms. The treatment is generally aimed to increase the dopamine bioavailability, either by replenishing the dopamine precursors or by inhibiting the breakdown of dopamine. The mainstay of treatment during early phases is the administration of dopamine replacement agent levodopa (also called L-dopa), which is the precursor to dopamine. Unlike dopamine, levodopa can cross the blood–brain barrier and can convert to dopamine in the brain. However, the conversion of levodopa in the periphery nervous system can result in off-target effects. Hence, the combination of levodopa and dopamine decarboxylase inhibitor such as carbidopa or benserazide is commonly used to prevent the peripheral depletion of levodopa before it crosses the blood–brain barrier and enters the brain. Good symptomatic relief can be observed in PD patients with levodopa treatment at the early phase of disease progression, of which the treatment response is used as a criterion in PD diagnosis [7]. However, the therapeutic efficiency deteriorates as the disease progresses with continued loss of dopaminergic neurons in the substantia nigra. Several complications, such as motor fluctuations, “on/off” phenomena, and dyskinesias, are the common side-effects as a result of long-term levodopa treatment. These levodopa-related complications and disability have become a therapeutic challenge for late stage-PD patients [11].

On the other hand, under normal physiological conditions, dopamine can be degraded by 3 enzymes: 1. the monoamine oxidase (MAO), which converts dopamine to 3,4,dihydroxy phenylacetic acid; 2. catechol-o-methyltransferase (COMT), which converts dopamine to 3-methoxytyramine; and 3. aldehyde dehydrogenase (ALDH). Dopamine can also be uptaken by the presynaptic neuron terminal through the plasma membrane dopamine transporter (DAT) or by the synaptic vesicles through vesicular monoamine transporter (VMAT)-2 (Figure 1B) [12]. Hence, MAO inhibitors or COMT inhibitors are also commonly prescribed by neurologists to synergistically increase the dopamine concentration in the brain. Notably, many of the enzymes involved in the biosynthesis and metabolism of dopamine are shared with other monoaminergic neurons. For example, TH and AADC are also involved in the biosynthesis of all catecholamine neurotransmitters, and AADC, VMAT-2, MAO, and ALDH are involved in the metabolism of serotoninergic (5-HT) neurons. This suggests that inhibiting these enzymes may also affect the functionality of other types of neurons and thus confer unwanted side-effects. Other therapies such as neuropsychiatric drugs can help with non-motor symptoms [13] or deep brain stimulation, which is used as an alternate therapy for advanced PD patients. 

Nevertheless, given the limitations of the current therapies and the fundamental pathogenesis of PD due to the loss of specific SNc mDA neurons, cell replacement therapy could be a promising approach to halt or reverse the progression of the disease.

## 3. Cell Therapy

In the late 80s and early 90s, several open-label uncontrolled trials involving 400 patients were conducted using human fetal midbrain tissues [14,15]. However, the outcomes were variable. In some cases, the grafts integrated into the host brain, restored dopamine release, reinnervated striatum, and ameliorated the clinical symptoms of motor dysfunction. In some promising cases, patients could be withdrawn completely from L-dopa treatment after transplantation (see the reviews in References [16,17]). However, two randomized, double-blind transplantation with human fetal midbrain tissue conducted in 2001–2003 revealed rather the opposite. No difference between transplanted and sham-operated patients was observed, but about 15% of transplanted patients developed moderate graft-induced dyskinesias [18]. This event was attributed to the presence of serotonin neurons in the grafted ventral midbrain tissue [19,20]. Failure to reproduce the therapeutic benefit from fetal graft transplant was also reported by other similar trials (for more details, see the review in Reference [14]). Challenges such as unresolved ethical concerns in terms of using fetal tissue from aborted human embryos, limited graft quantity (each transplant requires a sacrifice of 3–4 human embryos to obtain significant improvement in motor function), and high variability in therapeutic efficacy further preclude its feasible use in clinical application [14,17,21]. Thus, interest has shifted to produce mDA neurons from cultured stem cells for transplantation. To date, many types of stem cells have been employed to attempt regeneration of midbrain TH^+^ neurons. Among the stem cells which are commonly used are the adult neural stem cells (NSCs), mesenchymal stem cells, and the pluripotent stem cells.

### 3.1. Adult Neural Stem Cells

In the 1960s, the presence of NSCs was identified, which challenged the dogma that the adult brain is unable to regenerate and form new neurons [22]. Human adult NSCs have successfully been isolated from the biopsies of the hippocampus or subventricular zone of the lateral ventricles, where most neurogenesis happens (see the review in Reference [23]). The isolated neural cells can be maintained and expanded to form 3-dimensional free-floating clusters termed “neurospheres” in the presence of growth factors, such as epidermal growth factor (EGF) [24] and basic fibroblast growth factor (bFGF) [25]. Neurospheres were found to consist of neural stem cells and some more restricted neural progenitors that can differentiate into more specific neurons, astrocytes, and oligodendrocytes [26]. This finding prompted researchers to explore the generation and differentiation of midbrain TH^+^ cells from adult NSCs; some of them employed the use of cytokines such as interleukin-1 [27] or angiotensin II [28] or by overexpression of specific transcription factors involved in the development of mDA neurons (like Nurr1, Ngn2, Pax3, Wnt5a, and Lmx1) [29,30,31]. Yet, challenges to access and obtain NSCs that reside deep in the brain, the rapid loss of differentiation ability after long-term culture in vitro [32], and low graft survival [33] hamper its clinical feasibility and application. Furthermore, the cellular heterogeneity nature of NSCs complicates the reproducibility and functional interpretation after transplantation. These limitations make NSCs a less ideal candidate for treating PD.

### 3.2. Mesenchymal Stem Cells

Mesenchymal stem cells (MSCs) are multipotent, stromal cells with the ability to self-renew and differentiate into three common lineages such as osteocytes, adipocytes, and chondrocytes. The high availability, easy isolation from various tissues, proliferative characteristics, and immunity privileged with less ethical concerns make MSCs an attractive candidate for regenerative therapy (refer to Reference [34] for more details). Some studies showed that MSCs isolated from bone marrow [35], Wharton’s jelly or olfactory mucosa [36], and placenta [37] could also trans-differentiate toward ectodermal lineages like neuronal-like and glial-like cells [38,39] by exposing the cells to neural induction medium containing bFGF and EGF in additions to key neuro-morphogens such as retinoic acid (RA), ascorbic acid (AA), Sonic hedgehog (SHH), and FGF8. Systemic administration of MSCs showed that the cells crossed the blood-brain barrier; migrated to SNc and striatum; and then secreted multiple neurotrophic factors, cytokines, and exosome to protect dopaminergic neurons loss in PD models [40,41]. Furthermore, motor improvement was also evident from autologous transplantation of MSC-derived mDA neurons after 8 months in parkinsonian macaques [42], demonstrating its therapeutic potential in treating the disease.

One of the most promising studies by Trzaska et al. reported that mDA neurons could be generated from adult human bone marrow-derived MSCs, with an efficiency of ~67%. These cells expressed postmitotic midbrain markers PITX3 and NURR1, capable of releasing dopamine and showing electrophysiological properties [43]. However, a conflicting report demonstrated that low dopaminergic differentiation was observed in MSCs with poor graft survival after transplantation [44]. Notably, the neural differentiation potential of MSCs varies depending on the tissue origin [45,46]. Furthermore, adult bone marrow MSCs isolated from an aged donor are greatly affected by the age and commonly coupled with impaired neuroectodermal differentiation potential [47]. Thus, more work is required to determine the best MSCs source and to standardize the production process to ensure the consistency of MSC neuro-transdifferentiation before it is useful as a cell replacement therapy for treating PD.

### 3.3. Pluripotent Stem Cells

Pluripotent stem cells are stem cells with the ability to develop cells of all three germ layers, namely the mesoderm, endoderm, and ectoderm, except the extraembryonic tissues such as the placenta. The typical example of pluripotent stem cells is the embryonic stem cells (ESCs). 

Embryonic Stem Cells (ESCs) are primitive, undifferentiated pluripotent cells derived from the inner cell mass of preimplantation blastocysts, which have the ability to self-renew and differentiate into three germ layers except for extraembryonic tissues [48]. They can be maintained under undifferentiated status in vitro using bFGF (human ESCs [49]) or leukemia-inhibitory factor (LIF) (mouse ESCs [50,51]) while maintaining a normal karyotype. Despite the considerable potential as a tool to unveil human development, the use of ESCs sparks ethical controversies as the isolation involved the sacrifice of human embryos. 

Nonetheless, the substitute of ESCs was first introduced in 2006, with the invention of the novel reprogramming technology [52]. These substitute cells are named the induced pluripotent stem cells (iPSCs), which are generated using mouse skin fibroblasts by ectopic overexpression of four pluripotency-related transcription factors: Oct4, Sox2, Klf4, and c-Myc [53,54,55]. Subsequently, several groups have improved the reprogramming method to generate iPSCs from various somatic cells, including peripheral blood mononuclear cells [56], hematopoietic stem cells [57], and NSCs [58]. Human iPSCs can be generated from the patient [59,60,61], conferring iPSCs superiority over ESCs in delivering autologous graft with less allograft rejection. These advantages of iPSCs make the personalized and generalized cell therapy conceivable for further clinical application. 

Because of its pluripotency and research advancement in neurodevelopmental biology, the first mDA neuron differentiation was performed in vitro, albeit with low efficiency. In the early stage, mDA neurons generated from ESCs were largely based on the formation of embryo bodies (EB) [62] or by co-culturing with mouse stromal cell lines, such as the PA6 and MS5 lines [63]. However, the differentiation efficiency varies in both mouse (11–90%) and human (10–80%) [64]. Recent breakthrough from developmental studies has demonstrated that midbrain DA neurons originated from the ventral midbrain floor plate (mFP), which can be identified by the co-expression of Fox2a and Lmx1a [64,65,66,67,68,69,70]. This finding enables more precise identification of the bona fide stem/progenitor cells that can give rise to authentic mDA neurons [70]. Such discovery has also led to an in vivo testing of primate PD models, of which transplanting the ESC-derived mDA neurons showed promising improvement in motor function [65,71]. Early-stage human trials have also been initiated using human ESCs-derived neurons in the US, Europe, and Japan [72]. Next, we will review and discuss the brain development in vivo and the key signaling pathways that direct mDA fate in vitro.

## 4. Developmental Process of the Midbrain and the Formation of mDA Neurons

The development of mDA neurons in vivo involves complex events from neurulation, proliferation, and differentiation of neuron progenitors; to migration, and the formation of synapse and neural circuits. Multiple signaling pathways and specific morphogens guide these events at each differentiation stage. Here, we will review the brain development in vivo that serves the principal of mDA neurogenesis, which is used as the foundation for driving mDA neurodifferentiation from stem cells in vitro. 

**Figure 2:**
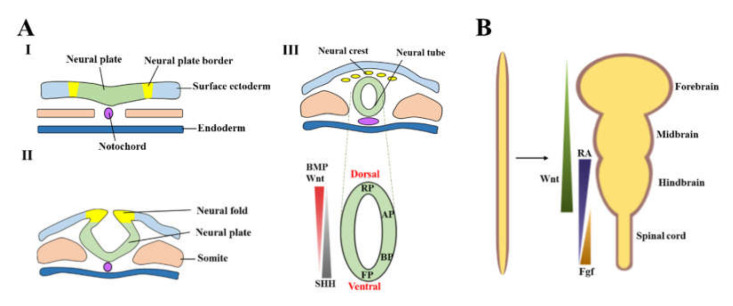
The schematic of the neural patterning principle: (**A**) Development of the neural tube (I-III) and the signaling governs the neural tube (which will develop into midbrain) at the D-V axis (III, bottom) and (**B**) the principle of neural development guided by the gradient signaling along A-P axis. Wnt regulates the development of the forebrain, midbrain, and hindbrain gradients of RA and Fgf involved in the segmentation of hindbrain and the spinal cord.

During the early development of the nervous system, neuroepithelial cells from neuroectoderm contribute to the formation of the neural plate, which then folds and fuse dorsally to form the neural tube, the primordium of the brain, and the spinal cord. This process is called neurulation (Figure 2A). After neurulation, the neural tube further develops into four main morphogenetic domains along the anterior-posterior (A-P, also called rostral-caudal) axis, namely the forebrain, midbrain, hindbrain, and spinal cord. These are primarily governed by Wnt, RA, and Fgf signaling (Figure 2). The floor plate (FP) is a crucial signaling center located at the ventral midline of the neural tube that extends from the spinal cord to the posterior diencephalon along A-P axis, whereas the roof plate (RP) is the domain located in the dorsal midline next to the neural crest, which gives rise to the peripheral nervous system. The basal plate and alar plate are dorsally and ventrally located in the lateral neural tube. Bone morphogenic protein (BMP) is dorsally secreted by the ectoderm and the RP, which directly opposes ventrally secreted SHH from the notochord and the FP (Figure 2 D) [73]. The dual morphogen gradients of BMP and SHH establish the dorsoventral axis and specify neuron populations along this axis. These precise spatial and temporal interplays of signaling specify the specific type of neural progenitors in each defined region [74,75]. The neural progenitor cells in the basal plate expand and migrate radially and tangentially into their target areas, extend axons and dendrites to form synaptic junctions with other neurons [76,77], and ultimately develop into lower motor neurons and interneurons [78,79].

The mFP and midbrain–hindbrain boundary (MHB) are two important signaling centers for mDA neuron development [80,81,82,83]. FP is an essential organizing center that is required for the patterning of the ventral neural tube. It is also crucial in guiding axon projections across the midline [84]. Recent studies have demonstrated that the most ventral region of the mFP has a distinct neurogenetic potential to produce mDA neurons, challenging its long perceived, non-neurogenic nature [82,85]. mDA neurogenesis is regulated via the SHH-FOXA2 signaling pathway. SHH induces FOXA2 expression in FP through its downstream target glioma-associated oncogene homolog (GLI) transcription factor that represses the expression of Dkk1, a Wnt/β-catenin inhibitor [86,87]. Gli positively regulates the downstream Ngn2 expression in the mDA progenitors and inhibits the basal midbrain transcription factors Nkx2.2 and Nkx6.1 [88,89,90,91]. Overexpression of Foxa2 also promotes mDA neuron formation and differentiation. Foxa2 heterozygous mutation study revealed a specific loss of SNc mDA neurons and abnormal motor behaviors shared with PD [90,92]. Moreover, FOXA1/2 directly inhibits the expression of Hairly and enhancer of Split 1 (Hes1) gene, which is essential for the generation of most midbrain GABAergic neurons located in the dorsal and ventrolateral midbrain [93,94]. On the other hand, FOXA2 also regulates SHH signaling by binding to SHH enhancer via a feedback mechanism [94,95]. However, FOXA2 is not uniquely expressed in midbrain since FP caudally extends to the hindbrain and spinal cord and involves the generation of hindbrain serotonergic neurons [96].

MHB (also termed isthmus) is a constriction formed near the junction between the posterior midbrain and hindbrain rhombomere 1, the most anterior region of the hindbrain, at embryonic day 9.5 in the mouse embryo and embryonic 4 weeks in the human embryo. MHB has a fundamental role in controlling both regional identity and neural identity of posterior midbrain and anterior hindbrain cells. Many transcription factors and signaling pathway ligands are key regulators related to MHB position, like Gbx2, Otx2, Fgf8, Wnt1, and En1/2. Coordinated expression and mutual repression of two transcription factors, orthodenticle homolog 2 (Otx2), which is mainly expressed in the forebrain and midbrain, and gastrulation brain homeobox 2 (Gbx2) in the hindbrain, is essential for the correct induction of the midbrain and hindbrain as well as the proper establishment of MHB. Ectopic expression of Otx2 or Gbx2 can alter the neural fate of the affected domain either to hindbrain or midbrain identity, respectively [97] (detailed in the Otx2 section). Two important morphogenic proteins secreted by the MHB, the FGF8 and Wnt1, are responsible for governing the neuron fate of the surrounding cells. 

Fgf8 is required for the formation of MHB. Its expression is maintained by Gbx2 and Wnt1 and is restricted to hindbrain rhombomere 1 [86,98]. A high level of Fgf8 in the hindbrain drives a hindbrain cell fate, while a low level of Fgf8 in the neighboring caudal midbrain directs cells toward a midbrain identity. These Fgf8-mediated events are achieved by regulating the Gbx2 and Otx2 expressions [97,99,100]. Altered Fgf8 level can shift the border of MHB and affect the size of mDA neurons region in caudal midbrain and serotonergic neurons located in the rostral hindbrain [101,102]. In mouse, Wnt1 is initially expressed in the entire midbrain anterior to the Fgf8 expression domain without overlap, but the expression becomes more restricted to the caudal midbrain adjacent to the Fgf8 expression domain at the late stage [103]. Studies also showed that Fgf8 expression in the MHB region was lost in Wnt1 knockout mice and cannot be reversed by forced ectopic Wnt1 expression in wild-type mice [104], suggesting that prominent Wnt signaling is required to regulate Fgf8 expression [105]. Additionally, Wnt1 also regulates the expression of midbrain transcription factors, like Otx2, En1/2, Nurr1, and Lmx1a [106,107,108]. In Wnt1 knockout embryos, the expression of En1/2 is lost in the MHB region [105], and Wnt1 inactivation also results in reduced progenitor proliferation, decreased viability of mDA neuron, and compromised MHB growth [103,105,109]. In β-catenin loss-of-function experiments, the key mDA neural progenitor genes such as Otx2, Lmx1a, Msx1, and Ngn2 were downregulated, and the midbrain neuron identity resembles the anterior hindbrain [105,110]. These alterations were also evident in Dkk1 null mouse embryos as a result of the overactivation of the Wnt/b-catenin pathway [105,111]. These findings confirm that Wnt signaling is vital in developing mDA neurons and in defining MHB fate through regulating Fgf8 signaling. 

## 5. Key Signaling, Transcription Factors, and Morphogens in mDA Neurodifferentiation

**Figure 3:**
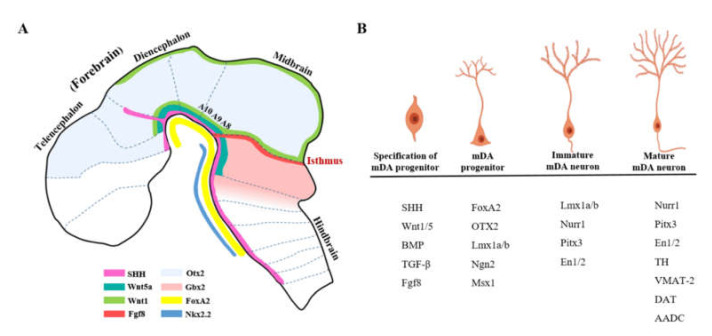
Signaling and morphogens involved in midbrain and mDA neuron development: (**A**) Sagittal plane of the brain, which illustrates the expression of the morphogens and transcription factors located in the midbrain and hindbrain, and (**B**) the main transcription factors expressed in the different stages of mDA progenitor or mDA neuron. (Adapted from Reference [112]).

**Figure 4:**
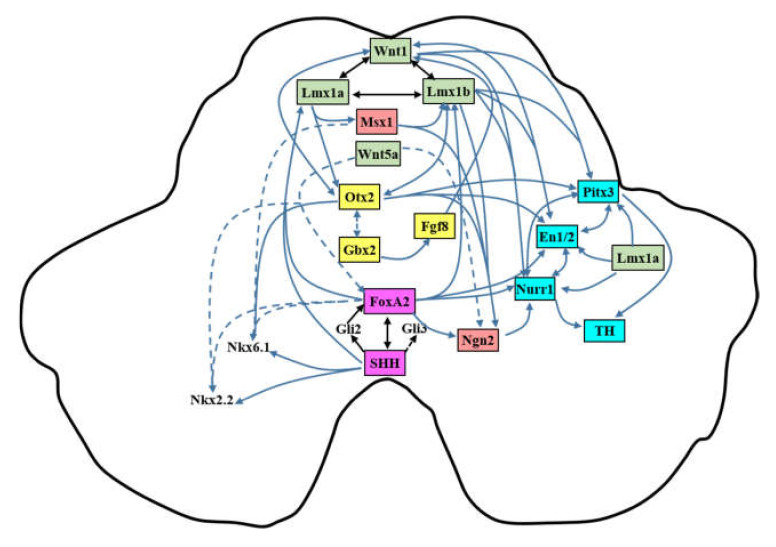
Genetic regulation of transcription factors in the development of the mDA neurons: The transcription factors are color-coded according to their expressions: purple = floor plate marker, yellow = midbrain–hindbrain boundary (MHB)-related genes, green = roof plate marker, red = neurogenesis marker, and blue = marker of postmitotic mDA neuron. The solid line denotes positive regulation; the dotted line means inhibition. FoxA2 is induced by Sonic hedgehog (SHH) either via SHH itself or its downstream Gli family (SHH-FoxA2 loop). FoxA2 regulates Lmx1a/b and suppress Nkx2.2 and Nkx6.1, while SHH positively regulates Nkx2.2 and Nkx6.1. Otx2 and Gbx2 mutually inhibit each other’s expression. Otx2 inhibits Nkx2.2 but positively regulates Nkx6.1. Gbx2 controls Fgf8 expression, which can regulate Wnt1. Wnt1 interacts with Lmx1a/b, forming a Wnt-Lmx1a/b loop. Lmx1a regulates Msx1 expression, which positively activates Lmx1b and pan-neuronal gene Ngn2 but inhibits Nkx6.1, while Wnt5a is an inhibitor for FoxA2 and Ngn2. FoxA2, Lmx1b, Lmx1a (via Msx1), and Otx2 all positively control Ngn2. Three postmitotic markers, Nurr1, En1, and Pitx3, reciprocally govern each other’s expression. Wnt1-Lmx1a/b, FoxA2 (absent in control of Pitx3), and Otx2 (absent in control of Nurr1) contribute to these three postmitotic markers. Nurr1 and Pitx3 facilitate the induction of tyrosine hydroxylase (TH).

Upon the onset patterning of neurulation, sequential activation of transcription factors prompts the differentiation of mFP cells to mDA neurons, during which the molecular identity of the immature mDA neural progenitors and the mature mDA neurons are established (Figure 3). In this section, we summarize the key signaling, transcription factors, and morphogens that are known to be implicated in controlling mDA neurogenesis (Figure 4).

### 5.1. LIM homeobox Transcription Factor 1 Alpha/Beta (Lmx1a/b)

Lmx1a and Lmx1b are LIM homeodomain transcription factors [113]. Lmx1a is initially expressed in ventral midline cells at E9 and then progressively expands dorsally. Lmx1b first appears in the midbrain since E8, but the expression confines to the roof plate, MHB, ventral mFP at E9.5. Both Lmx1a and b maintain their expressions until adulthood [114]. Lmx1a is essential for the specification of mDA neurons in the medial domain, whereas Lmx1b is responsible for lateral mDA neuron generation [115]. Lmx1a/b cooperatively regulate mDA neuron proliferation and differentiation with overlapping and cross-regulatory functions [116,117]. Lmx1a and Lmx1b are sufficient to induce the generation of mDA neurons through forced expression in the midbrain [82,93,118,119], and deletions of both can result in a near-complete loss of mDA neurons [115,117]. 

A study found that Lmx1a/b expression vanished in the Foxa1/2 knockout mutant [93]. Likewise, the gain-of-function experiment confirmed that Foxa1/2 induces Lmx1a/b expression. These suggest that Lmx1a/b functions cooperatively with Foxa1/2 to derive the FP fate [93]. Lmx1a/b controls the extent of neurogenesis by regulating the pan-neural protein Ngn2 directly or via repressing Ngn2 negative regulator Hes1. Furthermore, Lmx1a also regulates Ngn2 expression via another pan-neural gene named Msx1 (muscle segment homeobox homolog 1), which inhibits the lateral midbrain gene Nkx6.1, or inversely affects the Lmx1a/b-Wnt1 autoregulatory loop by targeting Lmx1b expression [118]. Lmx1a/b regulates Otx2 (a transcription factor expressed in forebrain and midbrain) expression through β-catenin, resulting in the downstream lateral midbrain transcription factor Nkx2.2 repression [116]. Alternatively, Wnt1 has the same regulatory mechanism on Otx2 either through the β-catenin complex or indirectly via the Lmx1b-Wnt1 autoregulatory loop [116]. Furthermore, the Lmx1b-Wnt1 autoregulatory loop directly regulates three key factors critical for the terminal mDA neuron differentiation and survival, like Nurr1, En1, and Pitx3. Deletion of Wnt1 results in defective expression of Lmx1a, Nurr1, and Pitx3 and a complete loss of mDA neurons [120,121]. Taken together, Lmx1a/b interacts with Wnt1, contributing to ventral mDA neuronal fate while suppressing the alternative lateral FP fate.

### 5.2. Orthodenticle Homeobox 2 (Otx2)

Otx2 is a transcription factor that plays a crucial role in normal neurogenesis and specification of ventral mDA neurons, which is mainly expressed in the forebrain and midbrain in the central nervous system [122]. In Otx2-knockout embryos, both the ventrolateral gene, Nkx2.2 and 5-HT, neurons are abnormally found in midbrain. Furthermore, the expressions of Ngn2 and Mash1 are severely reduced in the ventral midbrain cells of the Otx2-knockout embryos, causing significant loss of midbrain TH^+^ cells [122,123,124]. This means that the neurogenesis of mFP cells is lost and replaced by the cells with a hindbrain-like identity.

Additionally, the expression of Otx2 in forebrain and midbrain also forms an expression border adjacent to the anterior border of the Gbx2 expression domain, which defines the identity of rostral hindbrain [125,126]. The absence of Otx2 rostrally expanded the hindbrain Gbx2^+^ domain [122,125,127], while the knocked-in of Otx2 in rostral hindbrain caudally could shift hindbrain territory [97]. Mutual repression between Otx2 and Gbx2 is the key to proper regional segregation of caudal midbrain and rostral hindbrain, which are separated by the MHB organizer [127]. Inactivation of Otx2 rostrally reshaped the border of MHB, resulting in decreased expression domain of the midbrain related factors, such as Fgf8, Wnt1, and En1 [102]. Moreover, Otx2 represses the ventrolateral marker Nkx2.2 that directs serotoninergic neuron identity [122], while maintains the ventral midbrain Nkx6.1^+^ domain that is required to direct the basal plate progenitors to a ventral fate [128]. These suggest that Otx2 serves as a regulator for proper development and maintenance of mDA neuron identity partly by repressing the adjacent hindbrain fate [124].

### 5.3. Orphan Nuclear Receptor NURR1 (NURR1, also known as NR4A2)

Nurr1 is a transcription factor that belongs to the nuclear receptor superfamily, which is expressed in the mDA neurons in SNc and VTA at both the developmental and adult stages [129,130]. Nurr1 is vital in establishing dopaminergic phenotype in mDA neurons as it dictates the expression of TH, the enzyme which is responsible for dopamine synthesis. In Nurr1-deficient mice, the expression of TH was utterly absent during development, suggesting that the induction of TH in mDA neurons needs Nurr1 [131,132]. Multiple DA-related genes, such as AADC and ALDH2, were also found downregulated in Nurr1^−/−^ mutant mice, of which some expressions like DAT and VMAT were found completely vanished [133,134,135,136]. A study has demonstrated that these altered expressions were not attributed to the loss of mDA neurons but due to the Nurr1 deficit [133]. Nurr1 controls TH and DAT by binding its response element NBRE sequence in the 5′-untranslated region to their promoters [137]. Subsequently, the effect can be strengthened by its upstream regulator Wnt via binding of β-catenin to Nurr1 NBRE sequence [138]. A study has also shown that Wnt family member Wnt5a was found to act through Wnt/Rac1 signaling, to increase the number of Nurr1+ precursors, and to promote differentiation into TH^+^ dopaminergic neurons [31].

Nurr1 has also shown to increase the number of midbrain TH^+^ cells by forming Nurr1-RXR heterodimers with RXR receptors (a retinoid receptor) [139,140,141]. This suggests that Nurr1 works as a master regulator in the synthesis, storage, and metabolism of the dopamine neurotransmitter. Nonetheless, there is one report demonstrating that the expression of AADC in mDA neurons was independent of Nurr1 in vivo as the AADC expression was found present in Nurr1^−/−^ mice [133]. Similarly, the expression of other developmental regulators like En1/2, FoxA2, Lmx1b, and Pitx3 also remained in Nurr1^−/−^ embryos, with no developmental defects observed in the mice deficient. These findings indicate that Nurr1 has a lesser role in the early development of mDA neurons but is vital for its late survival and differentiation.

Notably, Nurr1 is also known to be involved in the pathogenesis of PD. Reduced Nurr1 expression [142] or Nurr1 mutation were commonly found in PD patients [143,144]. An in vivo study showed that Nurr1 activation could increase the number of TH^+^ cells in the SNc and improve dyskinesia [145]. Forced expression of Nurr1, together with Foxa2, was also found to relieve motor dysfunction in the PD mice [146]. Similarly, mDA neuron loss is ameliorated in PD mice when Nurr1 degradation is inhibited [147]. All these findings prompted a surge of interest to explore targeting Nurr1 as the therapeutic strategy for treating PD patients [148,149,150].

### 5.4. Engrailed Homeodomain Transcription Factors En1/2

En1 and En2 are homeodomain transcription factors that are expressed in cells in the rostral midbrain as well as in rostral hindbrain. En1 is highly expressed in all dopaminergic neurons in SNc and VTA, while En2 is only expressed in some of them [106]. En1 homozygous mutant mice died soon after birth and showed multiple developmental defects [151]. Although En2 mutation mice showed abnormal cerebellar phenotype, no alteration in the number of mDA neurons was observed [152,153,154]. In En1^-^/En2^-^ mutants, mDA neurons were completely absent in SNc and VTA, suggesting that En1 is essential for the survival of mDA neurons [155]. However, the decrease of mDA neurons in En1^+/-^ adult mice was antagonized by En2 recombinant protein infusion, suggesting that En1 and En2 have a biochemical equivalence ability in the development of midbrain [140].

En1 and En2 are transcription factors involved in the late-stage formation and maintenance of the MHB in addition to Otx2. En1 is known to control the proper landscape of dopaminergic neurons in the midbrain and serotonergic neurons in rostral hindbrain [156]. Knockout En1 in rodents caused caudal relocation of MHB, resulting in ectopic indistinguishable dopaminergic neurons and decreased serotonergic neurons in the rostral hindbrain [102,156]. It also forced ectopic expression of En1 in the midbrain, leading to the generation of ectopic dopaminergic neuron in the non-dopaminergic regions such as mFP [157]. In En1^−/−^ deficit mice, the expression of Ahd2, a subtype of ALDH enzyme responsible for RA production, which is important in patterning rostral mDA neurons, is completely lost [158]. The cholecystokinin (Cck), which marks the caudal VTA mDA subpopulation, however, is downregulated [159]. This causes the mDA neurons to lose their topographical identity [160].

En1/2 also interacts with Wnt1 in controlling the induction of MHB. Knockout Wnt1 could downregulate En1/2 expression in the MHB region [161]. En1 positively regulates Wnt1 by destabilizing β-catenin [162]. However, ectopic En2 expression in Wnt1 knockout mice rescued midbrain cell fates and Fgf8 expression [161]. These studies demonstrated that En1/2 functions as a downstream target of Wnt1 involved in the formation of MHB.

### 5.5. Paired-like Homeobox Protein (Pitx3)

Paired-like homeobox protein (Pitx3) is one of the postmitotic transcription factors expressed exclusively in terminally differentiated mDA neurons at E12.5 in addition to Nurr1 [163]. Pitx3 and Nurr1 are required for the maintenance of mature adult neurons [164]. The temporal expression of Pitx3 during development dictates the topographical fate of the resultant of mDA neurons in SNc and VTA. In *Pitx3* null mutant mice, TH^+^ neurons in SNc, but not in VTA, failed to develop and spontaneous locomotor activity was also affected [163]. This is because early Pitx3 expression specifies SNc mDA neurons prior to the expression of TH or otherwise results in VTA mDA neurons if the expression order of the two factors is reversed.

Pitx3 also controls midbrain rostral and caudal subset specifications of the Nurr1^+^ mDA neurons, through mutual regulation with En1. Pitx3 is initially induced by En1 to antagonize caudal characteristics by promoting Adh2 expression [158]. It can also directly repress En1 by regulating the transcription of genes encoding En1 modulatory proteins (Pbx1/3, Tle3) in the rostral midbrain. This results in the formation of Ahd2^+^ rostrolateral mDA neurons. In the Pitx3^−/−^ embryo, En1 expression is upregulated, driving mDA neurons to primarily Cck^+^ caudal fate [158]. On the other hand, Pitx3 expression diminished in En1^−/−^ embryo, which also downregulates Ahd2 expression. Nurr1 expression was found unaffected in both knockout models [158,160,165]. Hence, in En1^−/−^ embryo, only Nurr1-initiated mDA neurons with Ahd2^-^/Cck^-^ non-coded neurons were produced. This finding is similar to that of *Pitx3* and *En1* double-knockout model, of which both the expression of rostral marker Adh2 and caudal marker Cck are downregulated [160].

Additionally, Pitx3 functions to regulate TH expression in mDA neurons by partially binding to TH gene promoter at a bicoid-type binding element (5′-AAAGCC-3′), similar to Nurr1 [166]. Pitx3 targets Ahd2, an enzyme which produces RA, and increases TH and dopamine receptor 2, suggesting its role in regulating TH expression. In Pitx3^−/−^ embryos, the number of TH^+^ neurons in the rostral midbrain was reduced, but this could be salvaged by RA treatment [167]. These suggest that Pitx3 regulation of TH expression is RA signaling dependent. Moreover, both Pitx3 and En1 could synergistically potentiate Nurr1 in terminal mDA neuron differentiation by recruiting their co-repressor proteins and could release SMRT/HDAC (silencing mediator of retinoic acid and thyroid hormone receptor /histone deacetylase). This complex represses the Nurr1 target genes [158,165], therefore driving the mDA neurons to a more mature, differentiated phenotype.

### 5.6. Forkhead boxA1/2 (FoxA1/2)

FoxA1 and FoxA2 (FoxA1/2), the protein-coding genes of the forkhead/winged-helix family, are expressed in all ventral mDA neural progenitors and postmitotic mDA neurons [89,168]. FoxA1/2 are key regulators for mDA neurogenesis and specification. In FoxA1/2 heterozygous mutation, specific loss of SNc mDA neurons was observed and resultant abnormal motor behaviors were similar to that of PD [89,90]. In FoxA1/2 double knockout mutants, Ngn2 [89] and Lmx1a/b expressions were decreased [93]. Although Nurr1 and Pitx3 expressions were not affected by the absence of FoxA1/2 signaling [169], the mutant embryo did not survive beyond E9.5 [89]. A gain-of-function study confirms that FoxA1/2 induces Lmx1a/b expression and functions cooperatively to direct FP fate [93]. Moreover, FoxA1/2 knocked mice also decreased TH and AADC expressions as well as dopamine release in the dorsal striatum, which coupled with significant deficit electrophysiological properties [169]. This suggests that FoxA1/2 has a significant role in TH and dopamine biosynthesis and, so, the capability in driving the maturation of Nurr1^+^/TH^-^ and En1^+^/TH^-^ progenitors to form TH^+^ mDA neurons [18]. Notably, FoxA1/2 is not specifically expressed only in the midbrain, as FP progenitors also caudally extend to the hindbrain and spinal cord, and is involved in hindbrain serotonergic neuron development [96].

## 6. Midbrain Dopamine Neuron Differentiation from Pluripotent Stem Cells

Different strategies have been proposed to have driven successful mDA neurodifferentiation from pluripotent stem cells. These methods include a) direct fate conversion, b) the generation of the embryonic body (EB), c) coculture with neural-inducing stromal feeder-cell based method, and d) directed differentiation from pluripotent stem cells using growth factors/small molecules. In principle, all the mDA neurodifferentiation methods using pluripotent stem cells primarily share the three core differentiation stages: neuralization, specification, and maturation, except direct programming via ectopic overexpression of specific genes that bypass most of the development stages to turn cells into mDA neurons.

### 6.1. Direct Fate Conversion

Direct fate conversion, also known as direct cell programming, is a process to ectopically overexpress a set of specific genes to directly program somatic cells to differentiate or transdifferentiate to another cell type of the same or different lineage. Unlike reprogramming, the induced cells do not regain pluripotency or form intermediary progenitors but directly become the functional mature cells in the process of direct fate conversion. The first study was conducted back in 2010, which used the combination of three factors *Ascl1*, *Brn2* (also called *Mash1*), and *Myt1l* (also known as BAM or the Wernig factors); mouse embryonic and postnatal fibroblasts could be transdifferentiated into the induced neural progenitor cells (iNPCs) in vitro [170]. The same cocktails of transcription factors could also be used to convert human fetal and postnatal human fibroblasts to neural fate but with an additional gene called *NeuroD1* [171]. Likewise, studies had found that using BAM-converted iNPCs, when force expressed with *Lmx1a* and *Foxa2* [172], *Lmx1a*, *Lmx1b*, *Foxa2* and *Otx2* [173], or *Foxa2* and *Nurr1* [174], could also produce functional dopaminergic neurons at high efficiency. Another combination of transcription factors, *Lmx1a*, *Ascl1*, and *Nurr1* (LAN) [175] or together with *Foxa2*, *En1*, and *Pitx3* [176], have also demonstrated to have successfully induced the formation of mDA neurons from mouse and human fibroblasts, which possessed electrophysiological functions and dopamine release capability after integration into the brain of PD mouse in vivo [175,176]. The LAN, of which the Lmx1a was substituted with *Lmx1b,* was found able to turn 18% of mouse astrocytes to functional mDA neurons [177]. Nonetheless, extensive genetic manipulation and low reprogramming efficiency, survivability, long-term safety, and stability after transplantation remain unexplored.

### 6.2. EB-based Method

Lee et al. (2000) first described the method for generating midbrain TH^+^ cells from mouse ESCs using the following 5 steps: 1) expansion of undifferentiated ESCs; 2) embryoid body (EB) formation; 3) Nestin^+^ cell selection in a defined medium; 4) Nestin^+^ cell expansion with bFGF and mDA neurodifferentiation with SHH, Fgf8, and AA; and 5) withdrawal of mitogen to promote maturation of TH^+^ mDA neuron. This method could generate an average of 7 × 10^6^ TH^+^ cells from every 3 × 10^6^ ESCs, with an efficiency of >30% after expansion [62]. The differentiated TH^+^ neurons show synaptic properties and are capable of releasing dopamine. Though the protocol was established using mouse ESCs, it has also been adapted in differentiation using human ESCs and has successfully produced neural precursor cells with intermediate neural tube-like structure [178]. Nonetheless, the differentiation efficiency of this protocol is dependent on the EB size and the level of morphogens used in the protocol. Because of the nature of EB that involves the spontaneous formation and differentiation processes, this protocol often results in heterogeneous cell populations, which limits the efficiency of mDA neurons production [178].

### 6.3. Stromal Cell Coculture

Studies have found that MS5 [63] or PA6 stromal cells [179] can promote neurodifferentiation of mouse ESCs in co-culture. Takagi et al. further improved the differentiation method on PA6 cells by adding FGF20 together with FGF2, which yield 24% TH^+^ neurons out of TuJ1^+^ neurons with dopamine release [180]. The method has been improved, either by combining Noggin and Wnt5a at the early stage (day 1–5), with additions of FGF20 and FGF2 at the late stage (day 5–10), in PA6 coculture using animal ESCs [181] or by coculturing with immortalized midbrain astrocytes in the presence of SHH and Fgf8b [182]. This stromal cell coculture method produced TH^+^ neurons that were capable of releasing dopamine and showed electrophysiological properties in vitro. However, the nature of the soluble factors secreted by the stromal cells that facilitate neuronal differentiation in culture is unknown. Furthermore, this method generated TH^+^ neurons that are heterogeneous but not the defined homogenous mDA neuron derived from FP progenitor [67,183]. Moreover, these TH^+^ neurons poorly survived and integrated into the host brain when transplanted in PD [184] or even overgrow and generate undesirable progeny [182,185].

### 6.4. Guided Pluripotent Stem Cell Differentiation Using Growth Factor/Small Molecules and the Outcomes in Animal Trials

**Figure 5:**
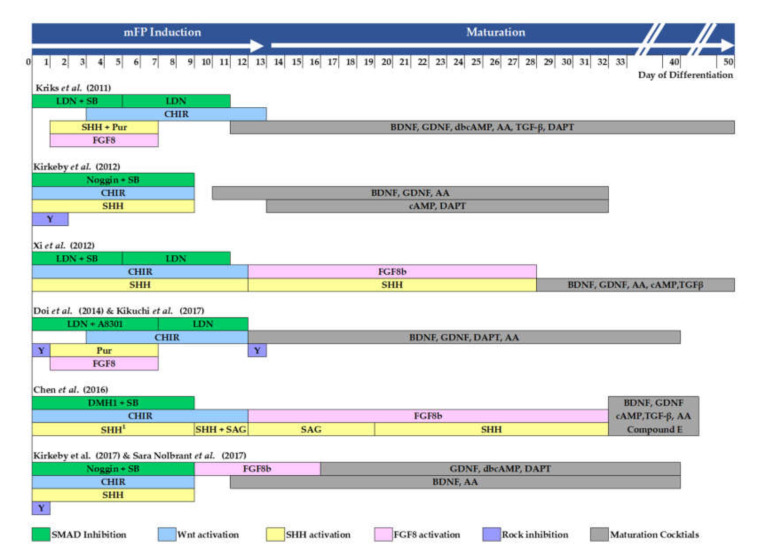
Current approaches to driving floor plate midbrain dopamine (FP-mDA) neuron differentiation from human pluripotent stem cells (PSC). Mothers against decapentaplegic homolog (SMAD) inhibitor: LDN193189 (LDN), SB431542 (SB), A8301, DMH1, and Noggin. Rock inhibitor: Y-27632 (Y); GSK3β inhibitor: CHIR99021 (CHIR); recombinant Sonic Hedgehog protein (SHH); smoothened agonist: purmorphamine (Pur), SAG; recombinant FGF8b protein (FGF8b); brain-derived growth factor (BDNF); glial cell-derived neurotrophic factor (GDNF); ascorbic acid (AA); dibutyryl cyclic adenosine monophosphate (dbcAMP) or cyclic adenosine monophosphate (cAMP), γ-secretase inhibitor: DAPT, Compound E; transforming growth factor β (TGFβ).)

Advances in pluripotent stem cell culture technology have enabled monolayer culture without feeder layers; directed differentiation of pluripotent stem cells (PSCs) can now be done in monolayer using combinations of growth factors and small molecules. This method is by far the most extensively studied and well-characterized through understanding the cues directly from developmental studies. Figure 5 illustrates the summary of the most current mDA neurodifferentiation protocol with a defined induction method and treatment timeframe, and the in vivo therapeutic outcome of the differentiated mDA neurons from each selected study are summarized in Table S1.

To induce early neuroectoderm differentiation from PSCs, the most recent method is to introduce dual- mothers against decapentaplegic homolog (SMAD) inhibition. This process inhibits two SMAD signaling pathways simultaneously, which was found to effectively prevent the development of mesodermal and endodermal lineages [186]. The two primary SMAD signaling pathways involve nodal and BMP signaling. Nodal signaling belongs to transforming growth factor-β (TGF- β) superfamily, and its inhibition was commonly achieved by small molecules SB 431542 or A8301, whereas Noggin [66], LDN-193189 [187] or dorsomorphin [188] are generally used in BMP signaling inhibition, be it in feeder-free monolayer culture [66] or three-dimensional EB culture [68]. However, the early Pax6^+^ neuroectoderm cells are generated with an anterior forebrain neural identity by default but can be re-specified to a posterior fate in response to caudal morphogens, like RA and Fgf8 [83,189,190]. Early exposure (day 11) to high levels of SHH (125–500 ng/mL) along with dual SMAD inhibition protocol increased FP marker FOXA2 at the expense of anterior neuroectoderm fate [83]. Moreover, prolonged SHH application beyond day 11 of differentiation did not affect FOXA2 expression while early application of caudal factors Wnts or GSK3β inhibitor (e.g., BIO 100 nM) or RA increased FOXA2^+^ cells [83]. However, SHH alone induced FOXA2^+^ ventral FP progenitors absent of midbrain characteristics.

Kriks et al. proposed an improved differentiation protocol to generate LMX1A^+^/FOXA2^+^ FP progenitors by using a potent GSK3β inhibitor CHIR99021 (3 µM on day 3 of PSC differentiation), a potent small molecule which is an activator of Wnt signaling [65]. The modulation of Wnt signaling was then studied further by Kirkeby et al., who concluded that CHIR99021 could pattern the neural progenitors from anterior to the posterior region in a dose-dependent manner and that the application of a lower concentration of CHIR99021 (0.7–0.8 µM) at an early stage of differentiation (from day 0) is more effective to drive PSCs to the midbrain fate [68] or otherwise results in caudal hindbrain fate at higher concentrations (>1 µM). Likewise, Xi et al. also found that temporal control of CHIR99021 (0.2–0.6 µM) application is crucial at early iPSC differentiation for patterning midbrain fate [69]. Interestingly, they also reported that the application of 3 µM CHIR99021 at day 3 of differentiation, as suggested by Kriks’s et al., failed to produce progenitors with midbrain identity using their protocol, probably due to some differences between the two protocols, e.g., basal medium and Matrigel [68]. Although Xi et al.’s protocol could produce mFP progenitors capable of developing into βIII-tubulin^+^ neurons, they failed to form TH^+^ dopaminergic neurons without late application of FGF8 (d12-d28) and a lower level of SHH (20 ng/mL) [69]. This suggests that the FGF8 signal is required to promote mDA neural progenitor differentiation. The differentiated FP-mDA neurons expressed most midbrain and A9 mDA neuron markers; exhibited neuro-electrophysical characteristics in vitro [69], capable of secreting dopamine; reversed amphetamine-induced rotation behavior; and engrafted with promising local innervation after transplantation in PD animal models from mouse, to rat and monkey [65,68]. The FP-mDA neurons-treated PD monkeys had survived for at least two years without any tumor formation [191].

In 2014, the Jun Takahashi team successfully generated Foxa2^+^/Lmx1a^+^ mDA neurons with an efficiency as high as 76% by using CORIN^+^ cells sorted at day 12 of differentiation from PSCs. About 18% of them expressed TH^+^ when administrated in a PD rat model [70]. They then generated the Corin^+^ FP-mDA neurons from PD patients and healthy individual iPSCs and found that the cells did not show mortality, tumor formation, or inflammation after transplanted in monkeys for 12–24 month-follow-up. This finding confirms the therapeutic efficacy and safety of the produced FP-mDA neurons [71]. Regardless of behavioral improvement in FP-mDA neurons-treated subjects, the outcome was lower than subjects receiving high-dose L-DOPA treatment [71]. This indicates that further optimization (e.g., cell dose and repeated injection) is required to obtain a more significant therapeutic outcome. Microarray analysis of transplanted mDA progenitors, comparing subjects that had excellent or relatively poor graft survival, revealed 11 upregulated genes, namely the *Ednrb, Cdh11, Crabp1, Rtl1, Dlk1, Pmel, Hapln1, Hist1h1a, Zic1, Crabp2,* and *P2rx3* [71]. These gene sets could be useful markers to define the mDA neuron subpopulation that was correlated with the outcome.

Recently, the Parmar group found that the common markers which are usually used to identify PSC-derived mDA neural progenitors, such as Lmx1a, Lmx1b, Foxa1, Foxa2, Otx2, Foxp1/2, and Msx1, were also expressed in the subthalamic nucleus (STN) neural progenitors, most of which are glutamatergic neurons [192]. Hence, the previous protocols which used these markers for assessing mDA progenitor identity may suggest the coexistence of both mDA neural progenitors and the more rostral glutamatergic neurons in the differentiated population [192,193]. In a retrospective analysis of more than 30 batches of human ESC-derived FP-mDA neurons transplanted in PD mice, they revealed that the expression of mDA progenitor markers Foxa2, Lmx1a, and Corin and the maturation markers TH, Nurr1, and AADC were not correlated with mDA neuron yield in vivo [194]. Instead, markers that are commonly expressed in the more caudal ventral midbrain and midbrain–hindbrain boundaries (MHB), like En1, Wnt1, SPRY1, CNPY1, FGF8, and Pax8, were found positively correlated with mDA neuron outcome in vivo. These cells showed a more mature A9-like morphology at the administered site in the striatum in rats. They demonstrated that caudal culture treatment with FGF8b at a late differentiation stage (day 9–day 16) reduced STN neuron contamination and increased mDA neuron differentiation [194].

In a nutshell, successful differentiation mDA neuron requires Foxa2^+^ progenitors, which can be generated from PSCs primarily through the SMAD/SHH signaling pathway and temporal control of the Wnt signaling. Specification of the intermediate Foxa2^+^ cells to mDA neurons is primarily governed by SHH and FGF8, albeit with differences between several key publications [82,85,195]. Furthermore, little progress is made to improve the maturation of the mDA neuron, of which the current method requires 30–50 days to complete the maturation process despite the varying level of TH^+^ cells.

## 7. Considerations for Autograft or Allograft

The debate on the choice of autologous or allogeneic cells for PD patients is ongoing, and it has yet to reach a consensus judging from the pros and cons and the clinical needs of which the autologous or allogeneic graft could offer. It is generally accepted that autologous cell source is preferred as it omits the need for immunosuppression and offers better cell engraftment. On the other hand, allogeneic cells are the more economical and readily available source for production but are limited by the rate of immune-rejection and low graft survival. This can also be seen from a transplantation study comparing allogeneic and autologous iPSCs-derived mDA neurons. As expected, the autograft revealed a better TH^+^ cell survival *in vivo* without the need for immunosuppression, while a significant immune reaction was observed in allogeneic transplantation with diminished iPSCs-derived TH^+^ cell survival *in vivo* [196]. Nevertheless, the use of autologous iPSCs-derived mDA might not be beneficial to patients with known PD-associated gene mutations. In this instance, allogenic cells from a healthy donor may be considered. Alternatively, with advances in gene-editing tools such as clustered regularly interspaced short palindromic repeats(CRISPR)/CRISPR-associated protein (Cas9) technology, it is possible either to correct the mutated gene in the autologous iPSCs or to remove the human leukocyte antigen (HLA) to generate a universal iPSC allogenic lines which could escape the immune recognition system in the host [197] (For more detail, please refer to the review in Reference [198]). Noteworthily, more evidence on the use of mDA neurons from the gene-edited iPSC line is required to ensure the long-term safety and efficacy after transplantation prior to use in clinics.

## 8. Conclusions

In this review, we summarized the important transcriptional processes and signaling required for proper development of mDA neurons, which serve as the fundamental ground to the recent advances in directing mDA neurodifferentiation from pluripotent stem cells in vitro at high efficiency. With promising preclinical findings that show long-term survival and improvement in PD functions as discussed earlier, a new initiative, GForce-PD gathering the academics from US, Europe, and Japan, has been established to test pluripotent stem cell-derived mDA neuron in human [72]. This signifies an important milestone in bringing cell therapy closer to bedside PD treatment in humans. Whilst awaiting the outcome of the ongoing clinical trials, further understanding of mDA neurodevelopment and the genetic stability post-transplantation and the possible signaling controlling the proliferation of mDA neural progenitors may help to strategize necessary in vitro conditioning and enable sustainable cell manufacturing, especially when an allogenic cell source is involved.

## Supplementary Materials

The following are available online at https://www.mdpi.com/2073-4409/9/6/1489/s1. Table S1: Summary of in vivo transplantation studies using iPSC-derived mDA neuron.
