# Supplementary material for: Development and Differentiation of Midbrain Dopaminergic Neuron: From Bench to Bedside
Source: Cells. 2020 Jun 18;9(6):1489. doi: 10.3390/cells9061489 (PMC7349799; doi:10.3390/cells9061489)
Supplement: Supplementary file 1 [file cells-09-01489-s001.pdf]

Table 1: Summary of *in vivo* transplantation studies using iPSC-derived mDA neuron.

Authors	Transplanted Cells	Animal model	Cell dose/ injection site	Immuno-suppression	*In vivo* observation			
					Graft innervation	Behavioral improvement	Graft size	Graft TH^+ cells
Kriks et al. 2011	Nurr1+ progenitors (d25)	6-OHDA-lesioned mice (immunodeficient)	150,000/striatum	-	-	Amphetamine-induced rotation	-	-
		6-OHDA-lesioned rat	250,000/striatum	Cyclosporine A	Striatum	Amphetamine induced rotation; cylinder test, stepping test	-	15,000 TH^+ cells
		6-OHDA-lesioned monkey	1,250,000/injection ;3 sites/striatum (caudate and pre-commissural putamen)	Cyclosporine A	-	TH fibers extend >3 mm into the host	-	-
Kirkeby et al.2012	FoxA2/Lmx1a 80% (d10, d16)	6-OHDA-lesioned rat	150,000-300,000/striatum	Cyclosporine A	Striatum, Amygdala and Snr	amphetamine induced rotation; cylinder test,	15-20 mm^3	-
Dio at el. 2014	Day 12 sorted CORIN+ cells (d28)	6-OHDA-lesioned rat	400,000/striatum (putamen)	-	Striatum	amphetamine-induced rotation	3.4 ± 2.9 mm^3	$6,747 \pm 2,341$ TH+ cells
Chen et al. 2016	EN1+/OTX2+, LMX1A+/FOX A2+ (day18)	6-OHDA-lesioned mice (SCID)	200,000/striatum	-	Caudate-Putamen, Nucleus Accumbens (Nac) And Olfactory Tubercle	Amphetamine-induced rotation; cylinder test, rotarod test, and spontaneous rotation test	-	6110 ± 254 -7555 ± 913 TH^+ cells
Kikuchi et al. 2017	day 12 sorted CORIN+ cells (d28)	MPTP monkey	4,800,000/striatua m (putamen)	FK506	Caudate Nuclei, Putamen, And Whole Striatum	Neurological scores	39.4 ± 21.2 mm^3	$64,000 \pm 49,000$ TH^+ cells
Kirkeby et al.2017	FOXA2+/LMX1 A/B+ (day 16, 90.4%)	6-OHDA-lesioned rat (athymic)	50,000-100,000/striatum	-	Dorsolateral Striatum And Prefrontal Cortex	Amphetamine induced rotation; cylinder test,	0.37 ± 0.07 mm3/100,000 cells grafted	$3,716 \pm 1,026$ TH^+ per 100,000 cells grafted
